# Determination on the Coefficient of Thermal Expansion in High-Power InGaN-based Light-emitting Diodes by Optical Coherence Tomography

**DOI:** 10.1038/s41598-017-14689-y

**Published:** 2017-10-31

**Authors:** Ya-Ju Lee, Chun-Yang Chou, Chun-Ying Huang, Yung-Chi Yao, Yi-Kai Haung, Meng-Tsan Tsai

**Affiliations:** 1 Institute of Electro-Optical Science and Technology, National Taiwan Normal University, 88, Sec. 4, Ting-Chou Road, Taipei, 116 Taiwan; 2 Department of Electrical Engineering, University of Washington, Seattle, WA 98195 USA; 3 Department of Applied Materials and Optoelectronic Engineering, National Chi Nan University, Nantou, 54561 Taiwan; 4 Department of Electrical Engineering, Chang Gung University, 259, Wen-Hwa 1st Road, Kwei-Shan, Tao-Yuan 33302 Taiwan; 5 Department of Dermatology, Chang Gung Memorial Hospital, Linkou, Taiwan

## Abstract

The coefficient of thermal expansion (CTE) is a physical quantity that indicates the thermal expansion value of a material upon heating. For advanced thermal management, the accurate and immediate determination of the CTE of packaging materials is gaining importance because the demand for high-power lighting-emitting diodes (LEDs) is currently increasing. In this study, we used optical coherence tomography (OCT) to measure the CTE of an InGaN-based (λ = 450 nm) high-power LED encapsulated in polystyrene resin. The distances between individual interfaces of the OCT images were observed and recorded to derive the instantaneous CTE of the packaged LED under different injected currents. The LED junction temperature at different injected currents was established with the forward voltage method. Accordingly, the measured instantaneous CTE of polystyrene resin varied from 5.86 × 10^−5^ °C^−1^ to 14.10 × 10^−5^ °C^−1^ in the junction temperature range 25–225 °C and exhibited a uniform distribution in an OCT scanning area of 200 × 200 μm. Most importantly, this work validates the hypothesis that OCT can provide an alternative way to directly and nondestructively determine the spatially resolved CTE of the packaged LED device, which offers significant advantages over traditional CTE measurement techniques.

## Introduction

The lighting-emitting diode (LED) is a revolutionary, compact, and energy-saving light source. It can directly convert electrical currents into radiative emission through the electroluminescence effect^1,2^ and has been widely used in many commercial applications, such as LCD panel backlighting^3–5^, optical telecommunications^6,7^, and general lighting^8–10^. As the lighting market has grown rapidly in recent years, the demand for high-power LEDs has become higher than ever. Generally, a high-power LED device includes an LED chip mounted on a ceramic submount for mechanical stability and low thermal resistance, a wire bonding or electrical interconnect layer for the connection of an anode/cathode on the LED chip, and the encapsulant resin for the protection of the LED chip underneath. During the operation of a high-power LED device, one of the most challenging issues is finding appropriate packaging materials for reliable thermal management. The considerable amount of heat produced around the junction area of the LED chip is transferred to the entire device, leading to the thermal expansion of the packaging materials, in particular, the encapsulant resin. This inevitably causes strain due to the large difference of expansion degree between the LED chip and the encapsulant resin, which results in a reliability problem, hindering the output performances and possible applications of high-power LED devices in automotive forward lighting, color sequential projection display, and city environment engineering^11–13^. Therefore, proper thermal management and reliable inspection of packaging materials are necessary to ensure high optical outputs and long maintenance times of the high-power LED device. The coefficient of thermal expansion (CTE) is a physical quantity that indicates the thermal expansion value of a material upon heating^14,15^. When the LED is operated under a high-current condition, the thermal stress becomes a critical issue. Thermally induced stresses caused by the CTE mismatch between the packaging materials and the LED chip (or between the substrate material and the ceramic submount) can lead to fatigue of the wire bond and solder ball on the LED chip and cause delaminations or cracks in the packaged LED device^16,17^. All these issues limit the reliability and stability performance of the LED device. Therefore, in terms of advanced thermal management, accurate and immediate determination of the CTE of a high-power LED device is extremely important.

The time-domain OCT (TD-OCT) technique was first invented by D. Huang *et al*. in 1991 for biomedical applications^18^. Based on an interferometer configuration with optical path modulation, the method can acquire depth-resolved information. However, the system sensitivity and the imaging speed of TD-OCT are limited^19,20^. Hence, Fourier-domain OCT (FD-OCT), including spectral-domain OCT (SD-OCT)^21,22^ and swept-source OCT (SS-OCT)^23,24^ have been developed to overcome the limitations of TD-OCT. Compared to TD-OCT, FD-OCT is able to retrieve depth-resolved information without optical path modulation in the interferometer. Currently, both SD-OCT and SS-OCT systems can provide a frame rate of up to hundreds of frames per second and a system sensitivity of greater than 100 dB. In the past decade, OCT has been widely adopted as an *in vivo* imaging modality that provides noninvasive, high speed, and three-dimensional (3D) construction mainly in the field of biological specimens, such as gastroenterology, cardiology, dermatology, oral mucosa, and ophthalmology^25–29^. Typically, OCT can provide high resolutions of 1–10 μm in both the axial and transverse directions and can achieve a penetration depth of ~2 mm. Although OCT has been widely utilized in the above-mentioned biomedical applications, very few studies have employed OCT as an inspection tool in the semiconductor industry^30–33^. Through the examination of 3D OCT images, the spatial distribution and changes in a sample structure can be identified, allowing optical inspection in many kinds of industrial products. In this study, we examined temperature-dependent and depth-resolved OCT images to determine the instantaneous CTE of the packaging materials of InGaN-based (λ = 450 nm) high-power LEDs. Typically, the CTE of a material is measured by using a thermomechanical analyzer (TMA)^16,34^, which provides a single value of the CTE based on a specific material undergoing a uniform temperature change. After cooling down to room temperature, the change in the length of the material is measured to determine the CTE. However, the CTE of a packaged LED device is difficult to determine with the traditional method of using a TMA because it comprises many different constituent elements (materials) that must be measured at the same time. Therefore, it is important to develop an inspection tool that is capable of providing the spatial distribution of the measured CTE of the encapsulant materials, as it may have a strong connection with the stability and reliability of the LED device. As revealed in this article, OCT can not only examine the *in situ* variation of the CTE of packaged LED devices, but also reconstruct the measured CTE values into a two-dimensional (2D) spatial distribution over a given chip area (200 × 200 μm). The distances between individual interfaces in the OCT images were observed and recorded to derive the instantaneous CTE of the packaged LED device with different injected currents. The relationship between the junction temperature and the injected current was established using the forward voltage method. The results showed that the measured instantaneous CTE of polystyrene resin varied from 5.86 × 10^−5^ °C^−1^ to 14.10 × 10^−5^ °C^−1^ in the junction temperature range 25–225 °C, and exhibited a uniform distribution in an OCT scanning area of 200 × 200 μm. Most importantly, this work validates the hypothesis that OCT can provide an alternative way to directly and nondestructively determine the spatially resolved CTE of a packaged LED device, which offers significant advantages over traditional CTE measurement techniques.

## Results

**Figure 1:**
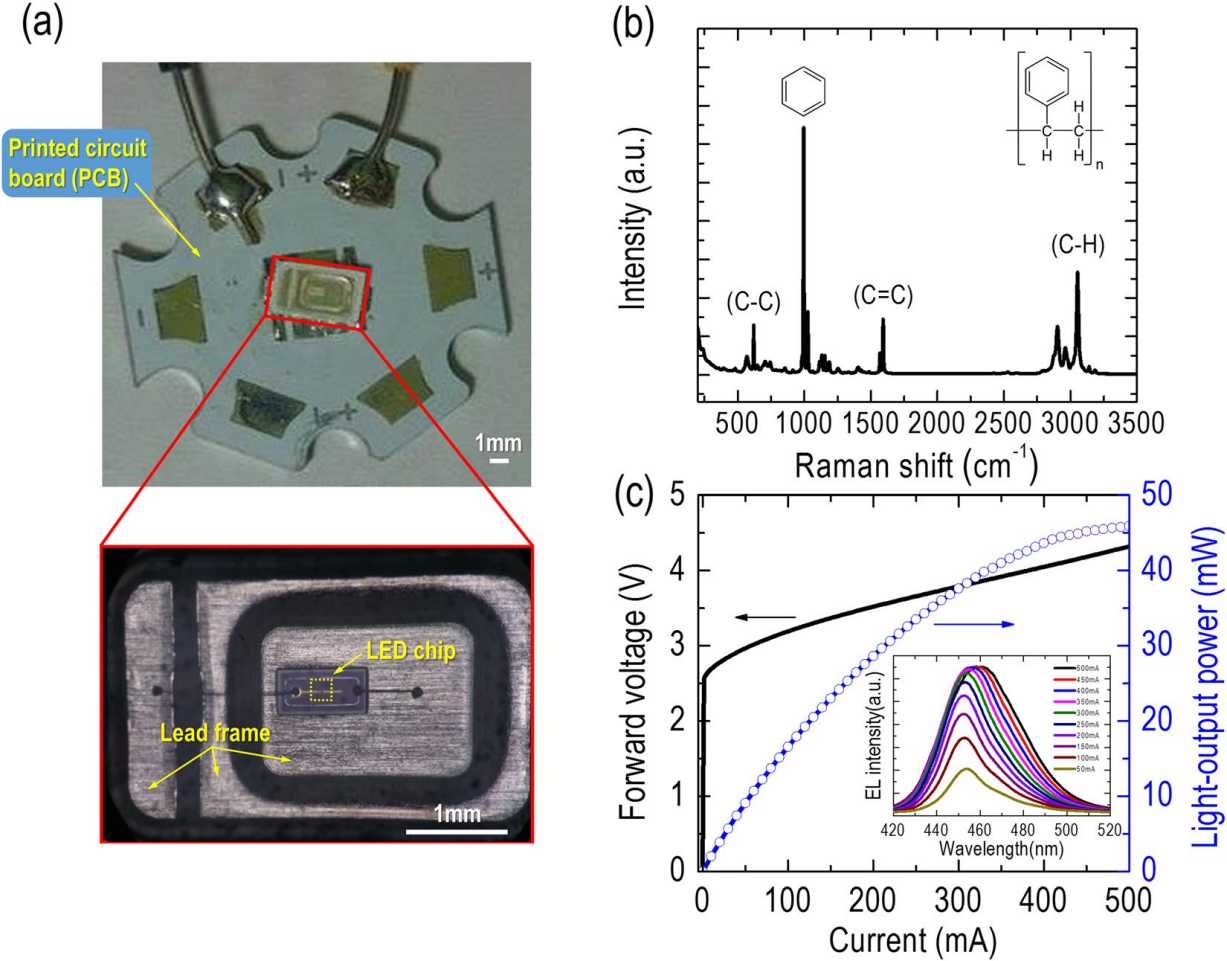
(**a**) Photograph of the packaged InGaN-based (λ = 450 nm) high-power LED used in this study. Inset: Microscope top-view image focusing on the center of the packaged LED device. The marked region (dash-line square) represents the chip area of 200 × 200 μm used for the subsequent statistical analysis of the spatial variations of the OCT images. (**b**) Raman spectrum of polystyrene resin excited by a 532-nm diode-pumped, solid-state laser. Inset: Repeating unit of the chemical structure of polystyrene resin. (**c**) Light-output power and forward voltage versus forward current for the packaged LED device. Inset: EL spectra of the packaged LED device under different injected currents (from 50 to 500 mA).

Figure 1(a) shows an image of the packaged InGaN-based (λ = 450 nm), high-power LED used in this study. A LED chip with dimensions 1.0 × 0.5 mm is mounted on a lead frame, and the anode and cathode of the LED chip are connected to the frame through gold wires. The lead frame is soldered on a printed circuit board (star shape) with a metal slug for the supply of injected currents. The inset of Fig. 1(a) shows a microscope top-view image focusing on the center of the packaged LED device. The wire-bonded LED chip mounted on the lead frame is clearly observed in the figure. In this study, polystyrene resin was used to encapsulate the LED chip underneath and form a flat-cavity geometry for its protection. This polymeric encapsulant generally exhibits high transparency, high refractive index, high temperature stability, and good hermeticity^35^. Figure 1(b) shows the Raman spectrum of polystyrene resin excited by a 532-nm diode-pumped, solid-state laser. The repeating unit of the chemical structure of the polystyrene resin is also illustrated in the figure. The polystyrene resin consists of a long-chain hydrocarbon in which alternating carbon centers are attached to phenyl groups. A dominant peak associated with the vibration of aromatic carbon rings in the polystyrene resin appears at approximately 1000 cm^−1^. In addition, two distinctive peaks, assigned to low carbon-carbon (C-C) and high carbon-hydrogen (C-H) vibrations, are clearly identified at around 600 cm^−1^ and 3000 cm^−1^, respectively. We can also observe a vibration of two carbon atoms with double bonds (C=C) that is stronger than that of the C-C single bond in the higher-frequency region of 1600 cm^−1^. The Raman spectrum confirms that the encapsulant material of our LED device was primarily composed of polystyrene, as no other dominant peaks are observed in Fig. 1(b). Figure 1(c) shows plots of the light-output power and the forward voltage versus the forward current of the packaged LED device. The turn-on voltage and series resistance of the packaged LED device were estimated by the Shockley diode equation to be around 2.66 V and 2.40 Ω, respectively^36^, comparable to that of typical InGaN-based high-power LED chips. The light output power of the LED increases gradually and is saturated at an injected current of approximately *I* = 400 mA, implying that a considerable dissipation of electrical-input power was induced in the form of unwanted heat. The electroluminescence (EL) spectra of the packaged LED device [inserts of Fig. 1(c)] are slightly blue-shifted (from 454.2 nm to 453.1 nm) at lower injected currents of I < 200 mA owing to the quantum-confined Stark effect, which is generally found in InGaN-based LEDs^37^. The EL spectrum exhibits a pronounced red shift (from 454.2 nm to 460.9 nm) at higher injected currents of I > 300 mA, confirming that considerable thermal heat was indeed induced and accumulated inside the LED chip.

**Figure 2:**
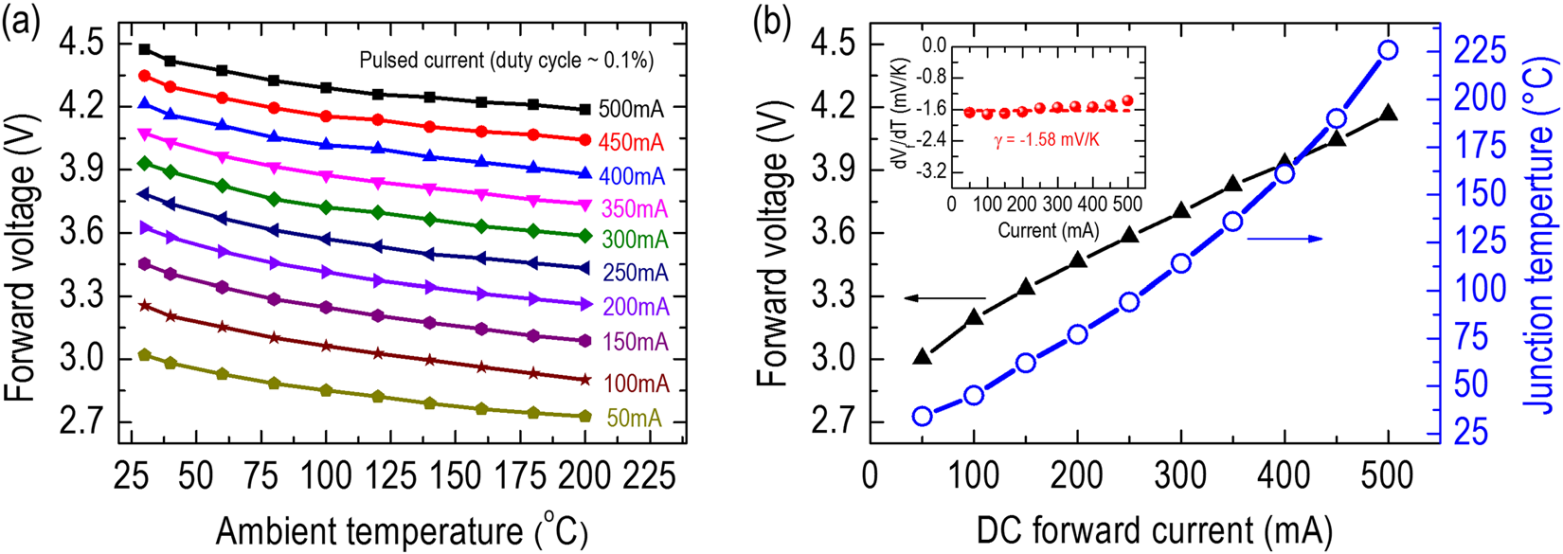
(**a**) Pulsed calibration measurement (duty cycle = 0.1%, pulse width = 6.5 μs), and (**b**) measured *V*_*f*_ and junction temperature versus injected current of the packaged LED device. Inset: Differential of *V*_*f*_ with respect to the temperature (*dV*_*f*_/*dT*) versus injected current.

**Figure 3:**
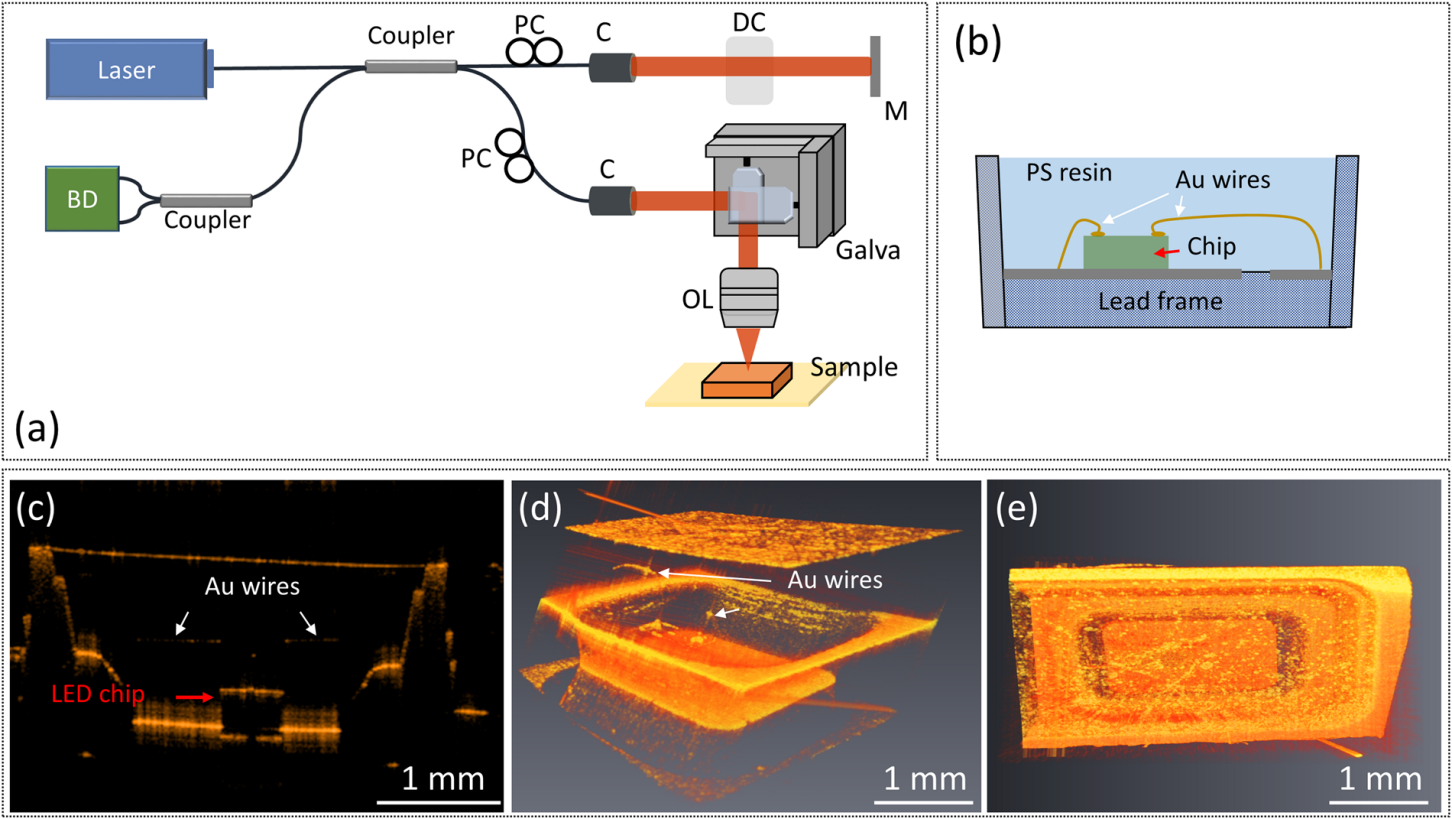
(**a**) Schematic diagram of the SS-OCT system. BD: balanced detector, PC: polarization controller, C: collimator, DC: dispersion compensator, OL: objective lens, M: mirror, and Galva: Galvanometer scanner. (**b**) Schematic of the configuration of the packaged LED device. (**c**) Cross-sectional 2D and (**d**) reconstructed 3D OCT images of the packaged LED device without an injected current. The locations of the Au wires and the LED chip are also labeled in the figures. (**e**) Top-view 3D OCT image of the polystyrene resin.

The dominant source of thermal heat is generated close to the active region of the LED chip, determining the operating temperature of the packaged LED device (generally referred to as the junction temperature). In this work, we measured the junction temperature by using the forward voltage (*V*_*f*_) method. Details about the implementation of the *V*_*f*_ method for the determination of the junction temperature can be found elsewhere^25,26^. Figure 2(a) shows the dependence of *V*_*f*_ on the ambient temperature (*T* = 30–200 °C) of the LED for different current levels (*I* = 50–500 mA). In this study, the ambient temperature was varied by placing the packaged LED device in a temperature-controlled plate, and the measurement was conducted in the pulse mode (duty cycle = 0.1%, pulse width = 6.5 μs) to minimize any possible thermal perturbation caused by a pulse and ensure that the junction temperature was equivalent to the ambient temperature. The calibration measurement plotted in Fig. 2(a) connects the junction temperature of the packaged LED device to its *V*_*f*_ for a range of currents. Figure 2(b) shows the *V*_*f*_ of the LED (the left-hand-side primary vertical axis) as a function of DC current in ambient room temperature. The differential of *V*_*f*_ with respect to the temperature for different currents is approximated by a constant *dV*_*f*_ /*dT* = −1.58 mV/K [insert of Fig. 2(b)], which is in agreement with experimental values reported in the literature^38,39^. The measured *V*_*f*_ and the calibration measurement displayed in Fig. 2(a) can establish the dependence of the junction temperature on different injected (DC) currents, as plotted in the right-hand-side secondary vertical axis in Fig. 2(b). Accordingly, the junction temperature of the packaged LED device increases rapidly with increasing injected currents and reaches a high value of *T* = 225 °C at *I* = 500 mA. Restated, such heat originating from the high junction temperature is likely to cause a serious issue related to the thermal expansion of the packaged LED device. Therefore, we used an SS-OCT system [Fig. 3(a)] to investigate the varied amount of thermal expansion in the packaged LED device under several current levels and determined the corresponding CTE values.

Figure 3(b) shows a schematic of the configuration of the packaged LED device tested in the SS-OCT system; the details of the constituent elements are provided in Fig. 1(a). Figure 3(c) shows a cross-sectional 2D OCT image of the packaged LED device without an injecting current. The lead frame (packaged cup), LED chip, and Au wires are clearly identified in the figure. Because OCT barely resolves images of materials with the same refractive index, we can only interpret the interfaces between the air and the polystyrene resin and between the polystyrene resin and the LED chip. Subsequently, the thickness variation of the polystyrene resin under different injected currents can be determined accurately. Figure 3(d) shows reconstructed 3D images of the packaged LED device without an injecting current, with the 3D scanning orientation perpendicular to the 2D image plane. Each 3D image comprises 500 slices of 2D images with a scanning interval of 5 μm between two consecutive 2D images. The constituent elements of the packaged LED device, such as the lead frame, LED chip, and Au wires, are clearly identified. Additionally, the surface morphology of the polystyrene resin is also well resolved [Fig. 3(e)], which is beneficial for the subsequent discussion about the spatial distribution of CTE values. The 3D OCT images shown in Fig. 3(d) and (e) suggest that OCT is a powerful solution for the exploration of both the internal structure and the external surface of the packaged LED device, which is difficult to achieve by traditional inspection tools, such as digital microscopes.

## Discussion

**Figure 4:**
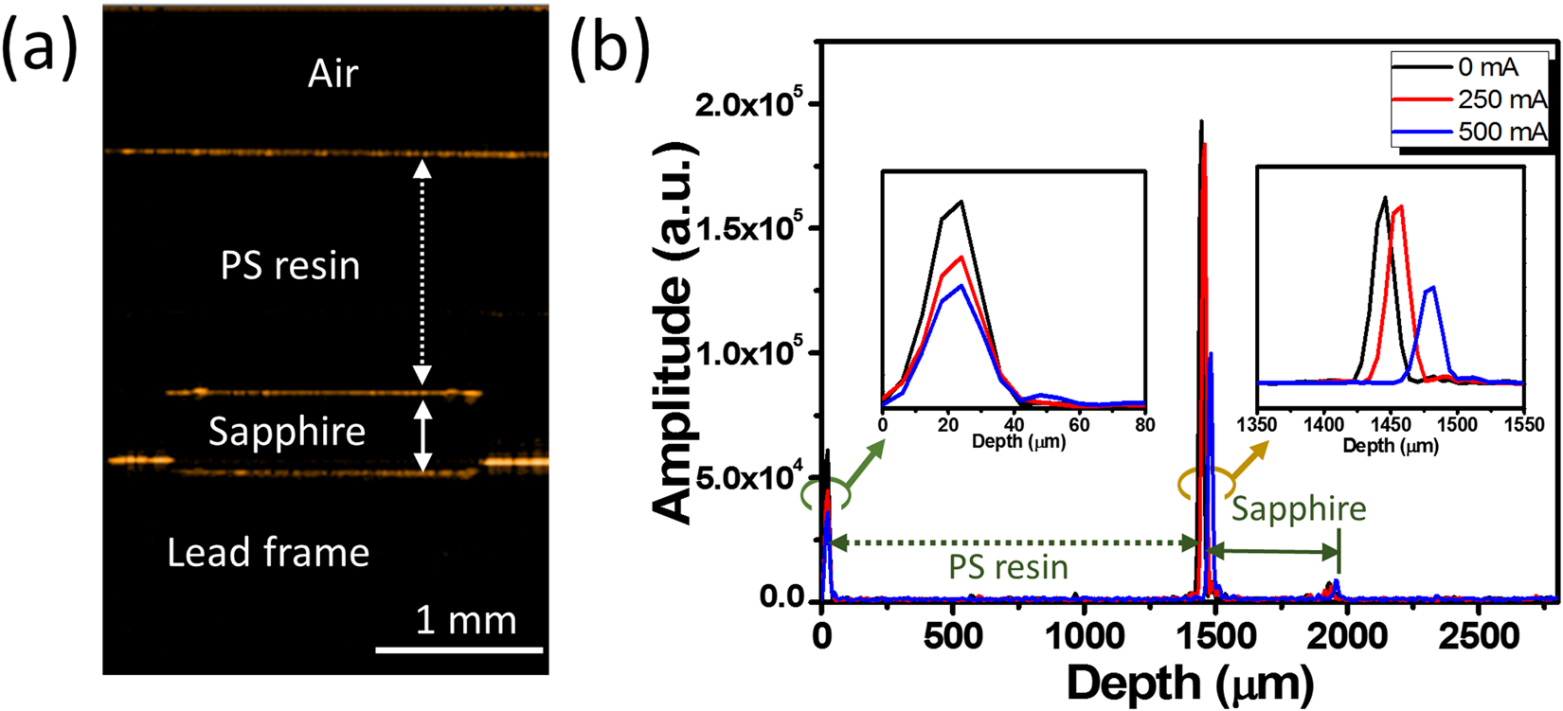
(**a**) Magnified cross-sectional 2D OCT image of the packaged LED device obtained near the LED chip area. (**b**) Depth-resolved OCT signal curves of the packaged LED device under different injected currents (*I* = 0/250/500 mA). Magnified images of the first (at 0 μm) and second (at ~1500 μm) peaks are also included in the figure to calculate the corresponding depth position shift under different injected currents.

To quantitatively examine the amount of thermal expansion of the polystyrene resin under different injected currents, we first obtained the cross-sectional 2D OCT image of the packaged LED device and magnified it on the LED chip area, as shown in Fig. 4(a). Because of the large difference in the refractive index at the hetero-material interface, a strong optical intensity was induced, which was attributed to backscattering of the incident laser; this led to an obvious brightness contrast in the scanned OCT image. In other words, the bright regions shown in the OCT image of Fig. 4(a) represent the hetero-material interfaces, which follow the sequence air/polystyrene resin, polystyrene resin/LED chip, and LED chip/lead frame from top to bottom. By measuring the distance between two adjacent interfaces, we can determine the extent of thermal expansion for a specific layer of the packaged LED device during the injection of the DC current. Figure 4(b) shows the depth-resolved OCT signal curves of the packaged LED device under different injected currents (*I* = 0/250/500 mA). The depth-resolved OCT signal curve can provide a general description of how the light propagates and reflects back through the entire packaged LED device. Accordingly, the first peak corresponds to the surface of the polystyrene resin, and it was hence defined as the starting point in the depth-resolved OCT profile for all the conditions with different injected currents. The second peak indicates the interface between the polystyrene resin and the LED chip, and its depth position shifts from 1446 to 1482 μm with increasing injected current from *I* = 0 mA to *I* = 500 mA [inset of Fig. 4(b)]. This suggests that a considerable amount of heat was accumulated in the polystyrene resin during current ramp-up, and this heat was mainly responsible for the observed thermal expansion. Similar thermal expansion was observed in the LED chip by simply comparing the separation shift between the second and third (which corresponds to the interface between the LED chip and the lead frame) peaks under different injected current conditions. Here, it should be noted that the total thickness of the epitaxial layers of the LED chip (including the p-type GaN, the multiple quantum wells, and the n-type GaN layers) was only about 4 μm, which is equivalent to the axial resolution of our OCT system. The thickness variation of the epitaxial layers caused by the increased junction temperature is difficult to determine, as it is typically smaller than the axial resolution of the OCT system. The observed thickness change of the LED chip was mainly attributed to the sapphire substrate under the chip, as the original thickness (without thermal expansion) of the sapphire substrate was 475 μm, much larger than the axial resolution of the OCT system.

**Figure 5:**
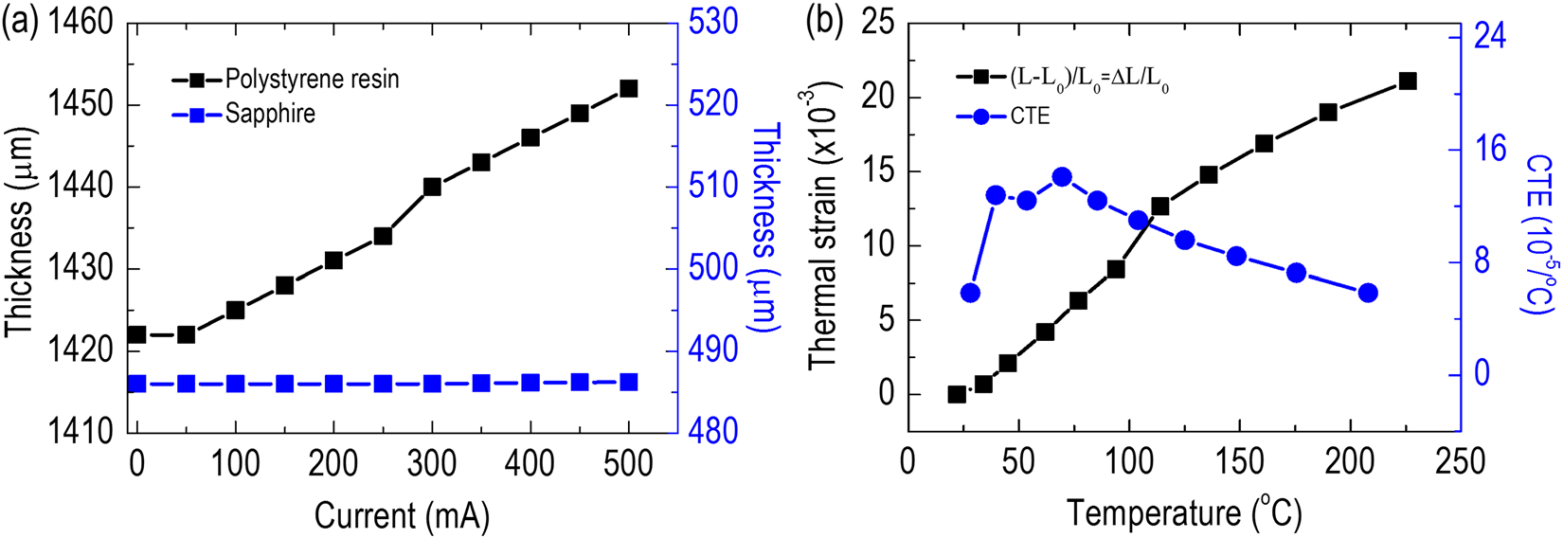
(**a**) Thickness variation versus injected current for the polystyrene resin (black squares) and sapphire substrate (blue squares). (**b**) Thermal strain (ε_thermal_) and instantaneous CTE versus junction temperature of the packaged LED device for the polystyrene resin.

Figure 5(a) presents the thickness variation versus the injected current, which was derived from the depth-resolved OCT signal curve of the packaged LED device for both the polystyrene resin (black squares) and the sapphire substrate (blue squares). The thickness of the sapphire substrate was barely changed, regardless of the variation of the injected currents. Generally, the level of thermal expansion depends on the bond strength between the atoms that form the materials. This result reflects the strong covalent bonds formed in the sapphire substrate (Al_2_O_3_), i.e., between aluminum and oxygen atoms, which lead to the high rigidity and low distortion of the substrate with respect to changes in the temperature. In comparison with the sapphire substrate, the thickness of the polystyrene resin is more sensitive to the variation of the injected currents and increases linearly with increasing injected currents. This implies that the atomic bond strength of the polystyrene resin (in particular, between carbon and hydrogen atoms) is much weaker than that of the sapphire substrate, which results in an obvious deformation of the polystyrene resin under a temperature gradient. The fractional increase of the thickness of the polystyrene resin was calculated by dividing the increased thickness (ΔL) by the initial thickness (L_0_) without an injecting current. (ΔL/L_0_) is also described as the thermal strain (ε_thermal_) because the change in the thickness of the polystyrene resin is mainly due to the elevated junction temperature associated with the increased injected currents. In this study, the thermal expansion was defined along the out-of-plane direction because the measurement of the in-plane expansion was confined and constrained by the lead frame package. Additionally, possible deformation of the polystyrene resin induced by the thermal strain caused by the thermal conductivity difference between the polystyrene resin and the LED chip can be neglected, as the thickness of the polystyrene resin (above 1 mm) was much larger than that of the LED chip heat source (~4 μm). Thus, it is difficult to generate strain-induced deformation on the polystyrene resin under such circumstances. Consequently, by exploring the dependence of the junction temperature on different DC-injected currents in reference to Fig. 2(b), the instantaneous CTE at any given temperature *T*_*m*_ can be determined as follows^12^:

1 $$CTE=\frac{({L}_{2}-{L}_{1})/{L}_{0}}{{T}_{2}-{T}_{1}}$$

where *L*_0_ is the initial thickness of the polystyrene resin without an injecting current, which expands to *L*_1_ at *T*_1_ and then to *L*_2_ at *T*_2_, and *T*_*m*_ = (*T*_1_ + *T*_2_)/2. Figure 5(b) shows ε_thermal_ and the CTE of the polystyrene resin against the junction temperature of the packaged LED device. The measured CTE varies from 5.86 × 10^−5^ °C^−1^ to 14.10 × 10^−5^ °C^−1^ for a junction temperature range of 25–225 °C, in good agreement with previously reported values^40,41^. This suggests that the adoption of a polystyrene resin with such high CTE may not be appropriate for packaging applications of high-power LEDs and the development of advanced polymeric composites with a reduced CTE is necessary. It must be noted that in this study, we used the forward voltage method to establish the dependence of the junction temperature on the injected current mainly for the LED chip; however, there can be a vertical temperature gradient between the LED chip and the polystyrene resin. The ramping slope of the junction temperature (i.e., the LED chip temperature) with increasing injected current is generally larger than that of the polystyrene resin temperature^34^. Therefore, according to the definition of the CTE given in Eq. (1), the derived CTE of the polystyrene resin was underestimated and could be referred to as a low limit for the OCT system. This is because we assumed that the junction temperature dominates in the packaged LED device and does not induce obvious temperature gradients in the entire device. Additionally, we also measured the CTE of pure polystyrene resin by the OCT system, in which a temperature-controllable hot plate was used as a heat source to induce the thermal expansion (please see Fig. S2 in Supplementary Information). Since there is no lead frame package to sustain in-plane confinements for the pure polystyrene resin, the thermal expansion along the out-of-plane direction becomes less significant, and the obtained CTE is approximately an order of magnitude smaller than that of packaged LED device. Most importantly, a layer-by-layer inspection of OCT images over a range of junction temperatures provides a direct and promising way to quantitatively determine the CTE of the constitute elements of the packaged LED device with high accuracy.

**Figure 6:**
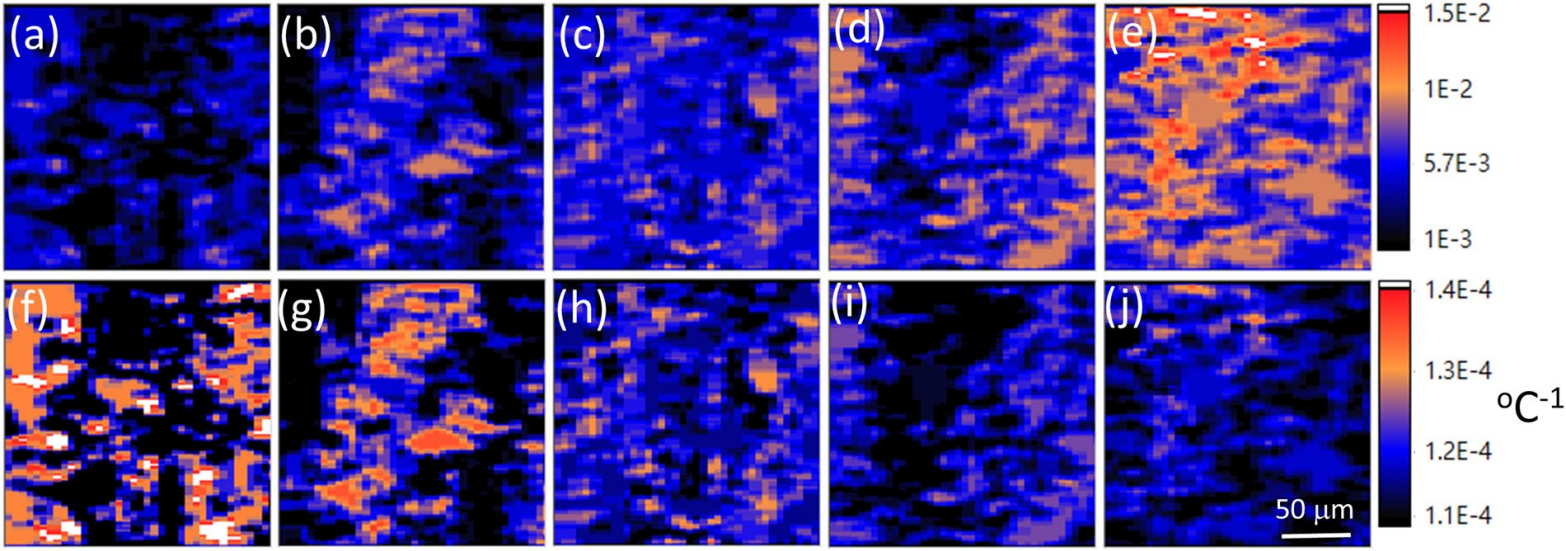
Spatial distribution of ε_thermal_ (**a**–**e**) and CTE (**f**–**j**) for the polystyrene resin over a specific chip area of 200 × 200 μm [marked in the inset of Fig. 1(a)] under different injection current conditions of 100, 200, 300, 400, and 500 mA.

Finally, to evaluate the spatial distribution of the measured CTE instead of determining its value at a single specific location, we scanned an area of 200 × 200 μm near the center of the LED chip using OCT [as marked in the inset of Fig. 1(a)]. Figure 6 shows the spatial distribution of ε_thermal_ and of the CTE of the polystyrene resin under different injection currents of 100, 200, 300, 400, and 500 mA. Figure 6(a–e), which were acquired by injecting different currents in sequence, show that ε_thermal_ is indeed increased with increasing injection current. Moreover, ε_thermal_ is defined as the ratio of thermal expansion (along the out-of-plane direction) to the initial thickness of the polystyrene resin, which is estimated by averaging the corresponding OCT images over a scanning area of 200 × 200 μm without current injection. Accordingly, the initial thickness of the polystyrene resin was set to 1423 μm for all injected current conditions. The results of Fig. 6(a–e) also suggest that the thickness of the polystyrene resin gradually increased with the injected current, and the thickness change reached a maximum expansion of 45 μm when a high current of 500 mA was applied. The results of Fig. 6(a–e) confirm that OCT can identify the ε_thermal_ value of the polystyrene resin with a two-dimensional spatial distribution. Figure 6(f–j) show the CTE distribution of the polystyrene resin near the center of the LED chip under different injected currents of 100, 200, 300, 400, and 500 mA. From these figures, the maximum CTE can be found when a low current of 100 mA is injected. When the injection current is increased to above 400 mA, the CTE values in the spatial distribution become smaller than those observed in lower current conditions. It must be noted that to increase the light extraction efficiency of InGaN-based LEDs, wet chemical etching is generally involved in the chip fabrication process to generate randomly roughened or textured semiconductor surfaces^42^. Figure S1 in the Supplementary Information shows a cross-sectional scanning electron microscope image of the top surface of the InGaN-based LED examined in this work. The hexagonal-cone features randomly distributed on the LED chip cause optical scattering of the swept laser of the OCT system with different degrees of diffusivity, which is mainly responsible for the observed fluctuation of the spatial variation of the measured ε_thermal_ and CTE values shown in the figure.

**Figure 7:**
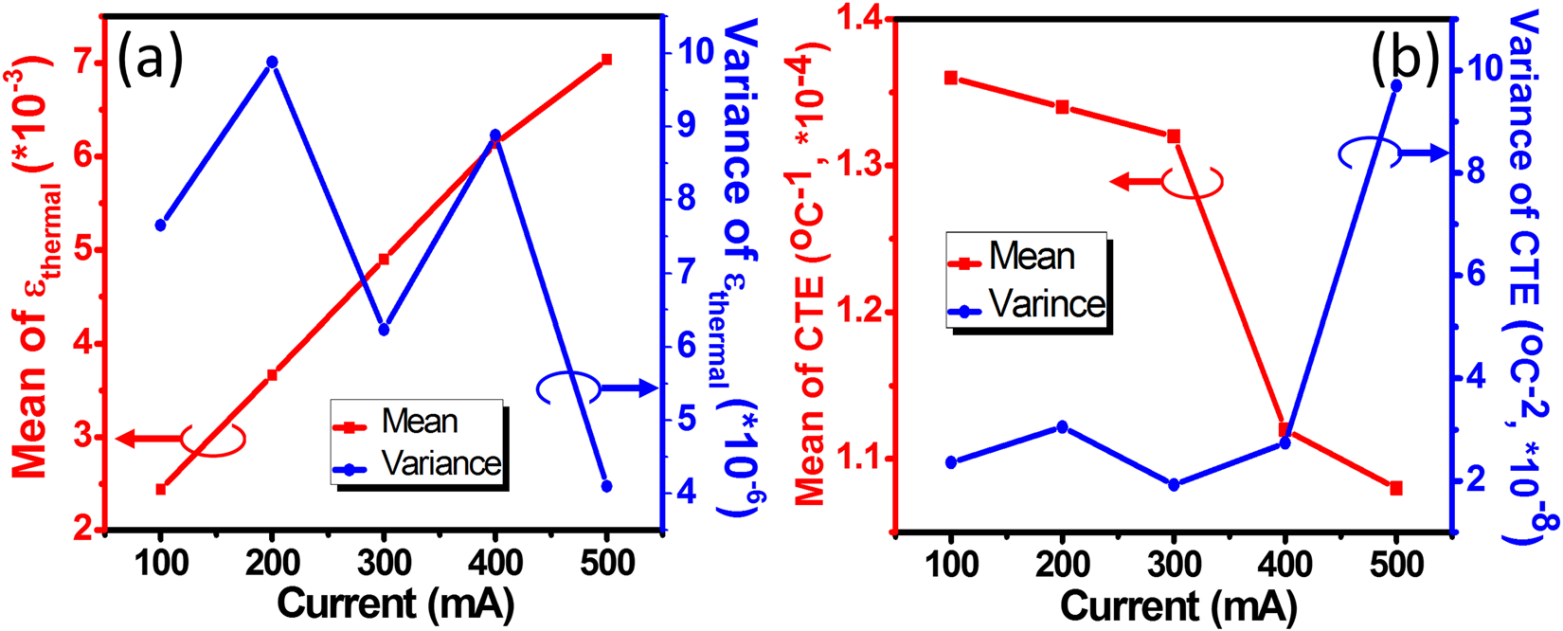
Statistical results (mean and variance) of (**a**) ε_thermal_ and (**b**) CTE values versus injected current for the polystyrene resin of the packaged LED device over a specific chip area of 200 × 200 μm [marked in the inset of Fig. 1(a)].

To further evaluate the spatial distributions of ε_thermal_ and the CTE of the polystyrene resin obtained from the OCT scans, statistical analysis was performed for a specific chip area of 200 × 200 μm [as marked in the inset of Fig. 1(a)]. Figure 7 presents the statistical results of the (a) ε_thermal_ and (b) CTE values against the injected current. When the injected current increases, the mean of ε_thermal_ is linearly increased [Fig. 7(a)], ranging from 2.44 × 10^−3^ to 7.04 × 10^−3^, and the corresponding variance of ε_thermal_ varies from 4.10 × 10^−6^ to 9.88 × 10^−6^. This indicates that the polystyrene resin used in this study was quite stable when the injected current/temperature changed and was uniformly deformed along the out-of-plane direction upon heating. Otherwise, we were not able to observe a linear increase of the mean of ε_thermal_ with increasing injected current (junction temperature). Similarly, the mean of the CTE [Fig. 7(b)] showed a stable performance when a low current was injected (100–300 mA) but slightly decreased when a current higher than 400 mA was applied. Accordingly, the mean of the CTE ranged from 1.08 × 10^−4^ °C^−1^ to 1.36 × 10^−4^ °C^−1^, while the corresponding variance varied from 1.92 × 10^−8^ °C^−2^ to 9.70 × 10^−8^ °C^−2^. Generally, the results indicate that OCT can be used to nondestructively observe the spatial distribution of ε_thermal_ and the CTE in the packaging materials of high-power LEDs and has potential applications in the evaluation of the reliability of packaged LEDs and in quality inspections of the device.

In summary, we used the advantages of OCT, such as nondestructive inspection, real-time visualization, layer-by-layer tomography, and three-dimensional reconstruction, to determine the CTE of an InGaN-based high-power LED packaged in polystyrene resin. Compared to the sapphire substrate, the thickness of the polystyrene resin was sensitive to the variation of the injected currents and increased linearly with increasing injected currents. The instantaneous CTE of the polystyrene resin varied from 5.86 × 10^−5^ °C^−1^ to 14.10 × 10^−5^ °C^−1^ in the junction temperature range 25–225 °C and exhibited a uniform distribution over a scanning area of 200 × 200 μm. We believe that the proposed methodology will contribute essential improvements in terms of inspection efficacy and measurement accuracy over traditional CTE measurement techniques.

## Methods

### Setup of the SS-OCT system

Figure 3(a) depicts a schematic diagram of the SS-OCT system used in this study. The center wavelength of the swept source (SSOCT-1060, AXSUN Technologies Inc., MA) is located at 1060 nm with a scanning spectral range of 100 nm. The optical beam from the light source is split into the reference and sample arms by a fiber coupler with a coupling ratio of 50/50. In the sample arm, a two-axis galvanometer (GVS302, Thorlabs Inc., NJ) is utilized for lateral and transverse scanning and the optical beam is then focused on the sample by a scanning lens (LSM02-BB, Thorlabs Inc., NJ). To reduce the dispersion resulting from the scanning lens in the sample arm, a dispersion compensator composed of N-SF8 glass is inserted in the reference arm. Finally, the return signal from both arms is received by a balanced detector (PDB460C, Thorlabs Inc., NJ) and digitized by a high-speed digitizer (ATS-9350, Alazar Technologies Inc., QC, Canada). To perform wavelength calibration before Fourier transform, the interference spectrum is resampled by an external clock signal from the swept source. The corresponding axial and transverse resolutions of the developed OCT system were approximately 4 μm and 7 μm, respectively. The measured system sensitivity of the developed OCT system was 103 dB. The developed OCT system can perform 2D or 3D scanning. Its physical scanning range covers a square area of 4 × 4 mm and the penetration depth can reach 2–3 mm, depending on the optical properties of the sample. The scanning rate of the light source was 100 kHz, which corresponds to a frame rate of 100 frames/s.

## Electronic supplementary material

Supplementary Information
