# Supplementary material for: Determination on the Coefficient of Thermal Expansion in High-Power InGaN-based Light-emitting Diodes by Optical Coherence Tomography
Source: Sci Rep. 2017 Oct 31;7:14390. doi: 10.1038/s41598-017-14689-y (PMC5663912; doi:10.1038/s41598-017-14689-y)
Supplement: Supplementary file 1 — Supplementary Information [file 41598_2017_14689_MOESM1_ESM.pdf]

Supplementary Information

Determination on the Coefficient of Thermal Expansion in High-Power InGaN-based Light-emitting Diodes by Optical Coherence Tomography

Ya-Ju Lee[1*], Chun-Yang Chou[1], Chun-Ying Huang[2,3], Yung-Chi Yao[1], Yi-Kai Haung[1], and Meng-Tsan Tsai[4,5*]

[1] Institute of Electro-Optical Science and Technology, National Taiwan Normal University, 88, Sec.4, Ting-Chou Road, Taipei 116, Taiwan
[2] Department of Electrical Engineering, University of Washington, Seattle, WA 98195, USA
[3] Department of Applied Materials and Optoelectronic Engineering, National Chi Nan University, Nantou 54561, Taiwan
[4] Department of Electrical Engineering, Chang Gung University, 259, Wen-Hwa 1st Road, Kwei-Shan, Tao-Yuan 33302, Taiwan
[5] Department of Dermatology, Chang Gung Memorial Hospital, Linkou, Taiwan
*yajulee@ntnu.edu.tw ;
mttsai@mail.cgu.edu.tw

Morphology examination of InGaN-based high-power LED device

Figure S1 shows cross-sectional scanning electron microscope (SEM) image of the top surface of the InGaN-based LED examined in this work. Accordingly, the hexagonal cones features are randomly distributed on the top surface of the LED chip, and their base and height are ranging from 200 to 600 nm. To increase the light extraction efficiency in InGaN-based LED, wet chemical etching is generally involved in the chip fabrication process to generate these hexagonal cones features. The hexagonal-cone features randomly distributed on the LED chip cause optical scattering of the swept laser of the OCT system with different degrees of diffusivity, which is mainly responsible for the observed fluctuation of the spatial variation of the measured $\varepsilon_{thermal}$ and CTE values shown in Fig. 6.

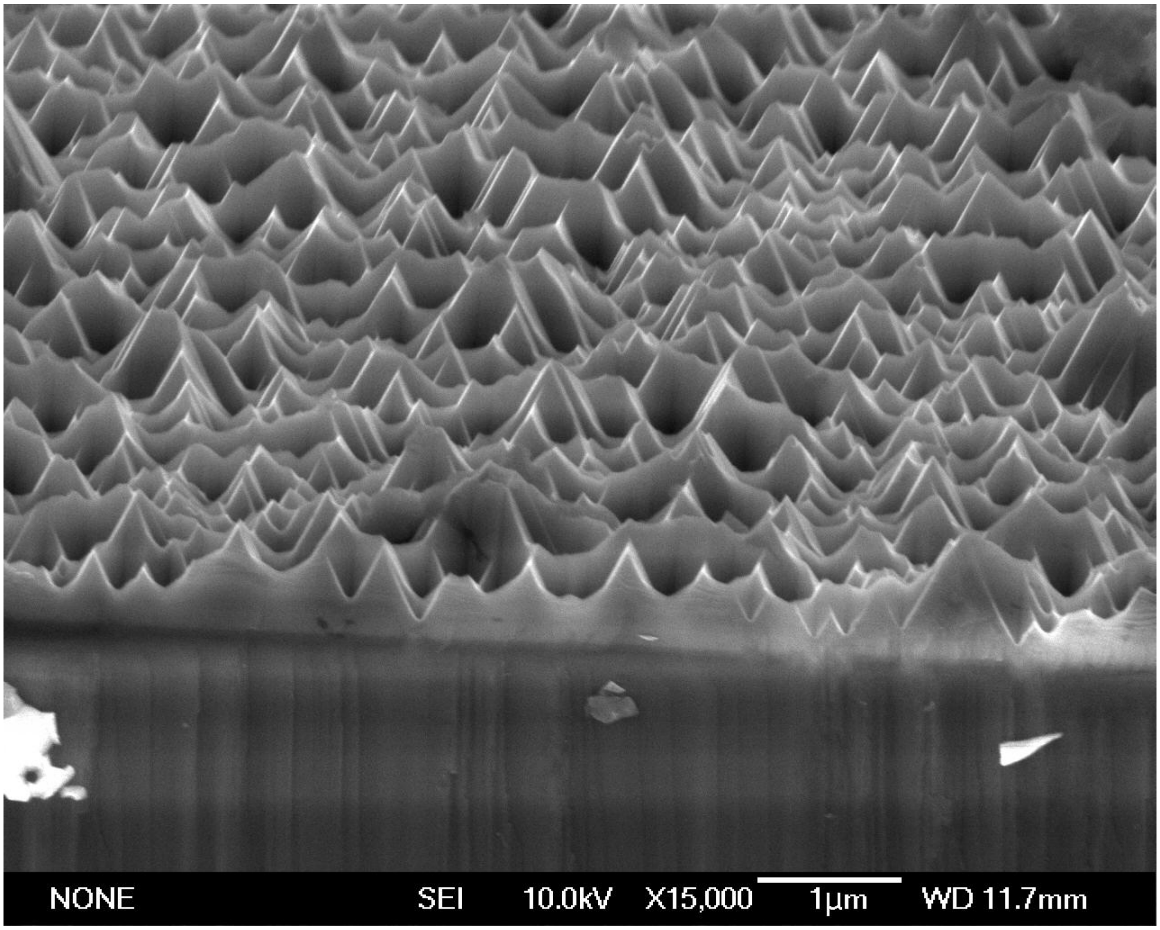

Figure S1. Cross-sectional scanning electron microscope (SEM) image of top surface of the InGaN-based LED examined in this work.

Measurement of the CTE of the pure polystyrene resin

In this experiment, the polystyrene resin is directly dispersed on the top surface of the glass substrate (5 cm × 5 cm), and then subjected to a 2-hour thermal curing process (in 150 ^{o}C oven) to crosslink the polymer chains and to form a rigid material, as shown in **Fig. S2(a)**. Unlike previous measurement on the packaged LED device, here we used a temperature-controllable hot plate as a heat source to induce the thermal expansion on the fabricated sample. After that, we repeated the OCT scanning procedures from T=30 ^{o}C to T=210 ^{o}C in a 20 ^{o}C interval, to derive the temperature-dependent CTE of the pure polystyrene resin. **Figure S2(b)** shows the CTE of polystyrene resin as a function of operating temperature set on the hot plate. In **Fig. S2(b)**, the averaged CTE and the standard deviation values shown as the error bars are obtained by averaging 1000 A-scans over a lateral distance of 1 mm. The CTE varies from 1.05×10^{-5} $^{o}C^{-1}$ to 1.42×10^{-5} $^{o}C^{-1}$ over a temperature range of 30–210 ^{o}C, and its dependence on the operating temperature exhibits a very similar tendency to that of the packaged LED device as shown in Fig. 5(b). As compared to the CTE of the polystyrene resin layer (5.86×10^{-5} $^{o}C^{-1}$ to 14.10×10^{-5} $^{o}C^{-1}$) in the packaged LED device, the CTE of the pure polystyrene resin obtained here is smaller by approximately an order of magnitude. It is because the pure polystyrene resin has no lead frame package to sustain in-plane confinements that the thermal expansion along the out-of-plane direction becomes less significant.

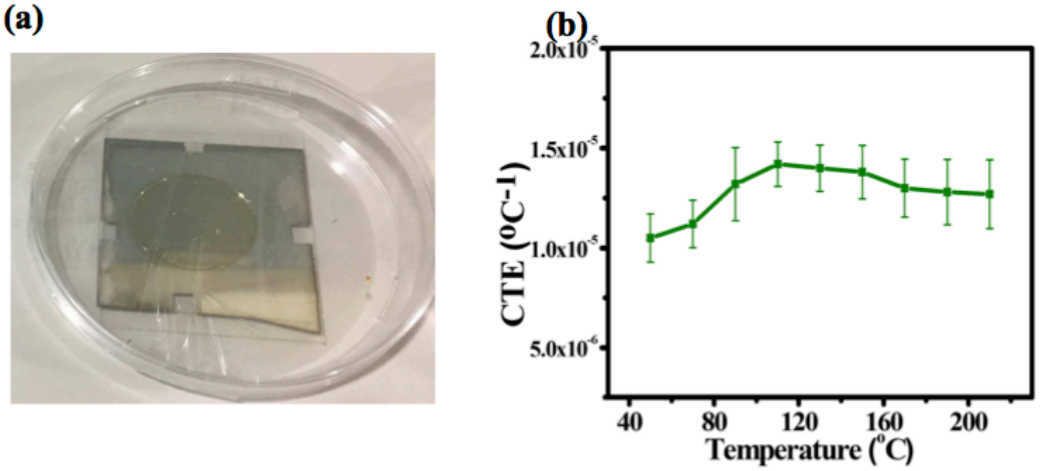

Figure S2. (a) Pure polystyrene resin dispersed on the top surface of the glass substrate as a reference material for the OCT scanning. **(b)** Instantaneous CTE against operating temperature for the pure polystyrene resin.
